# From Neighborhood to Household: Connections Between Neighborhood Vacant and Abandoned Property and Family Violence

**DOI:** 10.1007/s11524-024-00938-9

**Published:** 2024-11-14

**Authors:** Julia M. Fleckman, Julie Ford, Sophia Eisenberg, Catherine A. Taylor, Michelle Kondo, Christopher N. Morrison, Charles C. Branas, Stacy S. Drury, Katherine P. Theall

**Affiliations:** 1 Department of Social, Behavioral, and Population Sciences, Celia Scott Weatherhead School of Public Health and Tropical Medicine, Tulane University, 1440 Canal St. Tulane, New Orleans, LA 70112 USA; 2 Violence Prevention Institute, Tulane University, 1440 Canal St, New Orleans, LA 70112 USA; 3 School of Social Work, Tulane University, 127 Elk Place, New Orleans, LA 70112 USA; 4 School of Social Work, Boston College, 140 Commonwealth Ave., McGuinn Hall, Chestnut Hill, Newton, MA 02467 USA; 5 Philadelphia Field Station, Northern Research Station, USDA-Forest Service, 100 North 20th St., Suite 205, Philadelphia, PA 19103 USA; 6 Department of Epidemiology, Mailman School of Public Health, Columbia University, 722 West 168th St, New York, NY 10032 USA; 7 Department of Epidemiology and Preventive Medicine, School of Public Health and Preventive Medicine, Monash University, Melbourne, Australia; 8 Department of Psychiatry and Behavioral Sciences, Boston Children’s Hospital, 2 Brookline Place, Brookline, MA 02445 USA; 9 Department of Psychiatry, Harvard Medical School, Harvard University, 2 West, Boston, MA 02215 USA

**Keywords:** Family violence, Intimate partner violence, Child maltreatment, Vacant property, Neighborhood disorder

## Abstract

Rates of family violence, including intimate partner violence (IPV) and child maltreatment, remain high in the USA and contribute to substantial health and economic costs. How neighborhood environment may influence family violence remains poorly understood. We examine the association between neighborhood vacant and abandoned properties and family violence, and the role collective efficacy may play in that relationship. Data were used from a longitudinal cohort of 218 maternal-child dyads in a southern US city known for elevated rates of violence. Women were matched on their propensity score, for living in a neighborhood with elevated vacant and cited properties. Analyses accounting for clustering in neighborhood and matched groups were conducted to examine the association between neighborhood vacant and abandoned property and family violence and the potential mediating relationship of collective efficacy. The likelihood of experiencing child maltreatment at 12 months of age was more than twice as high for children living in neighborhoods with high vacant and cited property rates compared with women living in neighborhoods with fewer vacant and cited properties (OR = 2.11, 95% CI = 1.03, 4.31). Women living in neighborhoods characterized by high levels of vacant and cited properties were also more than twice as likely to report IPV (OR = 2.52, 95% CI = 1.21, 5.25). Associations remained mostly stable after controlling for key covariates. Collective efficacy did not act as a mediator in the relationship between vacant and cited properties and family violence. Reducing neighborhood vacant and cited properties may be an important target for interventions focused on reducing family violence.

## Introduction

Family violence—which includes intimate partner violence (IPV) and child maltreatment (CM)—is a global public health crisis contributing to increasing trends in violence and associated health and economic costs to society. In the USA, nearly half of women and more than 40% of men have experienced some form of IPV in their lifetime, including contact sexual violence, physical violence, and/or stalking [1]. For women, lifetime prevalence of physical violence by an intimate partner was an estimated 42%. Nearly one-third of women reported experiencing at least one act of severe physical violence by an intimate partner during their lifetimes. Additionally, nearly half of the women (49.4%) and men (45.1%) surveyed reported experiencing psychological aggression—the most common form of IPV—during their lifetimes [1]. Further, CM exacts enormous human and economic costs, threatening physical, mental, and behavioral health and contributing to epigenetic changes and altered brain and neurophysiological development in children [2]. According to the Children’s Bureau’s [3] summary of 2022 National Child Abuse and Neglect Data System (NCANDS) data, there were over half a million victims of child abuse and neglect, equivalent to a rate of 7.7 victims per 1000 children nationally.

A growing body of literature has demonstrated the influence of neighborhood context on neighborhood socioeconomic inequities, norms that promote violence and aggression, and various forms of violence. Findings that point to high levels of crime consistently remaining in neighborhoods with high poverty, few institutional supports, and weak community ties have led the shift toward efforts that critically examine neighborhood context [4], with a focus on the structural role of long-term disinvestment in creating areas of concentrated disadvantage—especially in urban areas [5]. Historically considered “behind closed doors” and thus often studied apart from other types of violence, IPV and CM are indeed associated with neighborhood context [6, 7]. Markers of neighborhood socioeconomic status have been strongly linked to both IPV and CM, including neighborhood deprivation, poverty, and unemployment status [7, 8]. Moreover, neighborhood structural disadvantage has been theorized to weaken social ties and drive household stress and alienation, not only increasing the potential for family violence but also leaving those impacted with fewer options for help [8, 9].

Neighborhood physical disorder, including vacant or visibly damaged housing or commercial buildings, litter or trash, and graffiti [10], can be considered a downstream effect and marker of this structural disinvestment and concentrated disadvantage [11]. Such visual cues of disorder, like vacant and code-cited properties, increase stress levels, negatively impact mental health, and reduce opportunities for building strong social ties with neighbors [12]—all factors thought to influence levels of both IPV and CM. Studies have also found associations between higher rates of IPV and CM with perceptions of neighborhood disorder [9, 11–14].

Collective efficacy, thought to influence levels of informal social control within neighborhoods and account for neighborhood-level variations in disorder [15], is understood as the presence of shared values and mutual trust among neighbors alongside a general willingness to intervene when socially unacceptable or violent behavior is witnessed [6, 15]. An association has been found between higher levels of collective efficacy and lower levels of street crime, and collective efficacy can mediate the effects of concentrated disadvantage and residential instability on violence [15]. Conversely, the relationship between collective efficacy and family violence has been less explored, with extant research yielding mixed resultse e.g. [13, 16, 17]. Despite some research demonstrating that neighborhood collective efficacy can act as a protective factor against IPV [13], additional research suggests collective efficacy does not appear to act as a strong mediator between neighborhood structural factors and IPV [17]. There may be important differences in how neighborhood social ties influence neighborhood violence versus IPV, where some forms of IPV might not be considered by neighbors as a community-level safety threat compared with other types of neighborhood violence [17]. Results have also been mixed regarding the relationship between neighborhood factors, collective efficacy, and the risks of CM [16, 18]. In particular, Emery et al. [16] questioned whether the well-documented relationship between collective efficacy and street crime can so easily be translated, suggesting community members may respond differently to public social problems versus matters considered private or within the family.

Yet, even within this relatively limited field of research, some common themes have emerged. Namely, levels of family violence, including IPV and CM, appear to be higher in neighborhoods facing concentrated disadvantage, long-term disinvestment, high levels of violent crime, and general signs of social disorder. Given that physical disorder is typically associated with concentrated disadvantage and higher levels of violence, identifying associations between levels of family violence and neighborhood physical disorder can add to the dearth of study in this emerging area. Further, the role of neighborhood processes, such as collective efficacy, in the relationship between neighborhood disorder and family violence has not been well explored and has ceded mixed results [6, 18]. The current study adds to the literature by examining the associations between neighborhood vacant and abandoned properties and family violence in a longitudinal cohort of maternal-child dyads in a southern US city known for elevated rates of community and family violence. Our hypothesis is that women living in neighborhoods with high rates of vacant and cited property will have an increased risk of family violence. Additionally, we examine parental stress and collective efficacy as potential mediators in the relationship between neighborhood vacant and cited properties and family violence. A deeper understanding of these relationships may inform the design and planning of neighborhood place-based interventions, such as improvements to built or natural environments, infrastructure, or amenities that may help reduce family violence.

## Methods

### Study Design and Sample

The current secondary analysis study was based on a sample of 395 mothers ages 18 to 43 years recruited from prenatal clinics, Women, Infants, and Children (WIC) clinics, and other ongoing University studies between years 2012 and 2017 in New Orleans, Louisiana. The community identification process, a mapping method to record epidemiologic indicators, was utilized to identify recruitment areas based on prevalence and incidence of community stressors. Individuals were excluded if they were less than 18 years of age, did not speak English, or were expecting more than one infant. The parent study for this secondary analysis examined the impact of cumulative maternal stress and attachment on birth outcomes and childhood physiological and behavioral outcomes. Mothers provided information about multiple levels of their and their infant’s social ecology using an interview-assisted computer survey administered face-to-face by a trained research assistant. Surveys were conducted at four time points: during pregnancy and when the child was 4, 12, and 18 months of age. This study was approved by the University Institutional Review Board.

The current study further restricted the sample to those participants who provided a complete residential address during pregnancy and who resided in New Orleans (*N* = 218), given that data on vacant and cited property exposure were only available within the city limits. Addresses were geocoded to link respondents to their residence and linked to their census tract or neighborhood. Of the 178 census tracts in New Orleans, 115 were included in analyses, with an average of 2 respondents per census tract (range = 1 to 6). The tracts represented are similar to most census tracts in New Orleans based on levels of concentrated disadvantage, vacant and abandoned property rate, employment, racial composition.

A propensity score analysis [19] was conducted to minimize selection bias or structural confounding, given the fact that neighborhoods with greater rates of vacant and abandoned property may also be more impoverished (which could drive the association with violence outcomes). The analysis examined overlap in the propensity for living in a neighborhood with high vacant and cited property rates vs. lower rates. Propensity models utilized individual and neighborhood socioeconomic indicators, including neighborhood concentrated disadvantage, a spatial clustering of economic and social disadvantage within neighborhoods [15], and level of urban life stressors, a measure of community life stressors [20], and maternal education, family annual income, age, and race and ethnicity. Mothers were then matched 2:1 (with replacement) on the propensity score (with a caliper of 0.05) (i.e., each woman with a propensity for living in a high vacant and abandoned property neighborhood was matched with two controls or women who had a lower propensity for living in such a neighborhood). Accounting for missing data in the outcomes, the final matched sample included 139 observations for examination of CM and 429 observations for examination of IPV.

### Measures

#### Outcomes

*IPV experiences of the mother* were assessed at baseline (during pregnancy), 4-, and 12-month follow-up for participants who reported being in a relationship with the *Hurt, Insult, Threaten, and Scream (HITS) Domestic Violence Screening Tool*. [21] The HITS screening tool is a validated, reliable four-item instrument that asks, “How often does your partner: physically hurt you, insult you or talk down to you, threaten you with harm, and scream or curse at you?” [20]. Participants are asked to give responses on a 5-point frequency scale: *(1) never*, *(2) rarely*, *(3) sometimes*, *(4) fairly often*, and *(5) frequently*. Answers to all four items are summed, resulting in scores ranging from 4 to 20. For the purposes of the present analysis, the variable was dichotomized so that participants who reported any abuse during pregnancy or after pregnancy were coded as (1) has experienced IPV and all other responses were coded as (0) has not experienced IPV.

*CM* was assessed via mother self-report using 20-items from three *Parent–Child Conflict Tactics Scale (CTS*) [22] sub-scales: psychological aggression, physical assault, and neglect. The measure was administered when the child was 12 months of age to gather how often in the past year the caregiver engaged in the assessed behaviors. Answer choices included: *(0) never*, *(1) once*, *(2) twice*, *(3) 3–5 times*, *(4) 6–10 times*, *(5) 11–20 times*, *(6) more than 20 times*, and *(7) not in the last year*. A positive report of any of the three types of behaviors in the past year (responses 1–7) was coded as 1, otherwise 0 (response 0).

#### Exposure

*Neighborhood vacant and cited property* was measured by the rate of vacant or cited property per 1000 population in the participant’s census tract or neighborhood at baseline (from 2012 to 2017), calculated based on publicly available data from the City of New Orleans. Rates were calculated and averaged across years of the cohort data collection. Properties included those with a judgment by the City’s Code Enforcement Division for lots that were unoccupied (usually coded as vacant with no structure) and that had one or more of the following violations: grass or vegetation growth higher than 18 in tall; trash, debris, or evidence of illegal dumping; and/or growth of noxious vegetation, such as poison ivy. The rate also included records of parcels with code violations, with an average of 28 violations per 1000 in a census tract (range = 0 to 165). In order to utilize propensity score matching which requires a binary exposure, the rate was also dichotomized based on the 75th percentile (38 per 1000 population) to characterize neighborhoods as having high vs. lower vacant and cited property rates.

#### Mediators

*Perceived collective efficacy* was examined as a potential mediator between neighborhood vacant and cited property and family violence outcomes. It was measured at baseline, 4 months, and 12 months using a validated 10-item measure, with five items aimed at measuring social cohesion and five informal social control [23]. Social cohesion items were measured on a 4-point scale (*0–3*) from *strongly disagree* to *strongly agree*, with respondents indicating the degree to which people in the neighborhood share the same values, are willing to help neighbors, and can be trusted, and that it is a close-knit neighborhood and gangs are not a problem in the neighborhood. Informal social control items were measured on a 4-point scale (*0–3*) from *very unlikely* to *very likely* with respondents indicating the likelihood that their neighbors would intervene if youth were skipping school and hanging out in the street, if youth were spray painting graffiti on local buildings, if children were showing disrespect to an adult, if a fight broke out in front of a house, and if the local fire station was threatened with budget cuts. Items were summed to create an index, ranging from 5 to 35, and averaged across timepoints.

#### Covariates

Several factors were also examined as potential confounders in the relationship between neighborhood vacant and cited property and family violence outcomes, and all were measured via self-report at baseline. *Sociodemographic factors* included maternal age, education, relationship status, race, ethnicity, and income. Race was based on self-reported, close-ended categorical questions that asked participants if they did or did not identify as a member of specific racial and ethnic groups, with the option of selecting more than one group. Race was first recoded into a categorical variable including all races identified and, given the sample distribution, subsequently recoded into three categories: Black, White, and Other. Marital status was a categorical question that included as follows: *(0) married, (1) married but live separately, (2) single/never married, (3) single/divorced, (4) widow, (5) in a committed relationship/never been married, and (6) in a committed relationship/also divorced or widowed*. Based on responses to the question on marital status, a dichotomous variable was created including (0) single and (1) married/in a relationship.

*Maternal exposure to adverse childhood experiences* was assessed using the *Adverse Childhood Experiences Survey (ACE)*, which was developed by Kaiser Permanente, in conjunction with the Centers for Disease Control and Prevention (CDC), as part of the Adverse Childhood Experiences Study [24]. Survey items examined a variety of hardships experienced during childhood prior to age 18 including abuse, neglect, and household dysfunction. Of the 26 items in this scale, 10 had binary response options including *(0) no* and *(1) yes*, and 16 had frequency of occurrence response options with a 5-point Likert scale (*(1) never, (2) once or twice, (3) sometimes, (4) often, and (5) very often*); these responses were also dichotomized (i.e., never = 0). A dichotomous variable was created in which participants who endorsed less than four items were coded as (0) and those that endorsed four or more were coded as (1). Prior studies have suggested an increase in poor health outcomes following report of four or more ACEs [24].

*Perceived stress* at baseline, 4 months, and 12 months was measured with the 4-item *Perceived Stress Scale* [25]. Respondents were asked how often in the last month they felt: “… that you were unable to control the important things in your life?”, “…confident about your ability to handle your personal problems?”, “… that things were going your way?”, and “… that difficulties were piling up so high that you could not overcome them?” [25]. Response options ranged from *(0) never* to *(4) very often*. The second and third items were reverse coded, and a summary score ranging from 0 to 12 was created for all items for each timepoint and then averaged across timepoints, with a higher score indicating increased stress.

*Anxiety* was also measured at baseline, 4 months, and 12 months with the *Rini Pregnancy-related Anxiety Scale* [26]. The 10-item scale with reliable internal consistency was designed specifically for use in pregnancy to assess the frequency of, or the extent to which, a woman worries or feels concerned about her health, the health of her baby, labor and delivery, and caring for the baby following delivery. Response options ranged from *(1) never or not at all* to (*4) a lot of the time or very much*. A summary score is created which includes reverse scoring the first and second item, and a mean score across time points was calculated. Scores range from 10 to 40, with higher scores suggesting increased anxiety.

### Statistical Analysis

Univariate, bivariate, and multivariate analyses were performed using SAS version 9.4, including generalized estimating equations (GEE) to take into account neighborhood clustering and conditioning on propensity score matched stratum or group. Standard errors, 95% confidence intervals, and, unless otherwise stated, a *p*-value < 0.05, were used to define statistically significant associations. Given the skewed distribution of data for both outcomes, logistic regression models analyzed dependent variables IPV and CM as binary outcomes (1 = reported IPV/maltreatment, 0 = no IPV/maltreatment). Vacant and cited property was coded both as a continuous measure (models 1 and 3) and dichotomized into high (75th percentile and above) and low vacant and abandoned property areas (models 2 and 4). Additionally, we examined the mediating role of collective efficacy. Potential mediation by collective efficacy was calculated utilizing the SAS MEDIATE Macro [27]. All covariates, including ACEs, anxiety, and stress, were included in final adjusted models.

## Results

Sociodemographic characteristics of the sample are presented in Table 1. The mean age of study participants was 26.8 years (range: 18 to 43 years). Similar to women more generally in New Orleans, most of the women identified as Black (65%) and over one-third (40%) had a high school education or less. Half of the sample (50%) had an annual household income of $24,999 or less. Over half (53%) reported being married or in a relationship. Approximately, 18% endorsed four or more ACEs, 46% reported at least one type of CM, and 36% reported experiencing at least one form of IPV. Mothers that lived in high (vs. low) vacant and cited property rate neighborhoods were significantly more likely to be younger, non-White, and single and have less education and income. No differences in collective efficacy, maternal ACE score, stress, and anxiety were identified between participants in high vs low neighborhoods.

**Table 1:** Characteristics of mothers at baseline, overall and by neighborhood vacant and cited property rate (*N* = 215)

Characteristics	Overall N	Overall %	High vacant and cited property rate (*n* = 54) *N* %		Low vacant and cited property rate (*n* = 161) *N* %		*p*-value^a^
**Maternal age**							.001
< 25	84	39.25	31	58.49	53	32.92	
25–30	54	25.23	14	26.42	40	24.84	
3135	47	21.96	5	9.43	42	26.09	
≥ 35	29	13.55	3	5.66	26	16.15	
**Maternal education**							.000
Less than high school	49	22.79	19	35.19	30	18.63	
High school graduate or GED	36	16.74	17	31.48	19	11.80	
Vocational/technical/associate degree	17	7.91	6	11.11	11	6.83	
Some college, no degree	41	19.07	10	18.52	31	19.25	
Bachelor’s degree or higher	72	33.49	2	3.70	70	43.48	
**Maternal self-reported race**							.002
Black	139	64.65	45	83.33	94	58.39	
White	58	26.98	5	9.26	53	32.92	
Other Asian	18	8.37	4	22.22	14	77.78	
**Household annual income**							.032
24,999 or less	101	50	31	63.27	70	45.75	
> $25,000	101	50	18	36.73	83	54.25	
**Maternal marital relationship status**							.000
Married or in a relationship	113	52.56	16	29.63	97	60.25	
Single, never married or divorced	102	47.44	38	70.37	64	39.75	
**Adverse childhood experiences**							.162
> 4	39	18.31	13	25.00	26	16.15	
< 4	174	81.69	39	75.00	135	83.85	
**Child maltreatment (yes)**	50	45.87	14	60.87	36	41.86	.104
**Intimate partner violence (yes)**	135	62.79	29	53.70	106	65.84	.113
		**Mean**		**Mean**		**Mean**	
**Average collective efficacy score** **(range 5–35)**	–	–	54	21.9	161	20.7	0.27
**Average perceived stress score** **(range 0–12)**	–	–	53	5.08	160	4.64	.345
**Average Rini score** **(range 10–40)**	–	–	49	16.78	148	17.33	.504

Based on non-missing values; less than 10% missing for each variable. ^a.^Likelihood ratio chi-square or Fisher’s exact or *t*-test

Table 2 presents both crude and adjusted results examining the association between neighborhood vacant and cited property rate and both CM and IPV. The likelihood of experiencing physical maltreatment, psychological maltreatment, or neglect at 12 months of age was more than twice as high for children living in neighborhoods with a high vacant and cited property rate (OR = 2.11, 95% CI = 1.03, 4.31, *p* < 0.05), increasing by 1% in the vacant and cited property rate per 1000 population; after controlling for key covariates, the adjusted rate was also statistically significant (aOR = 9.20, 95% CI = 1.03, 82.44, *p* < 0.05). Women living in neighborhoods characterized by high levels of vacant and cited property were also more than twice as likely to report IPV compared with women living in neighborhoods with fewer vacant and cited properties (OR = 2.52, 95% CI = 1.21, 5.25, *p* < 0.05), % in the vacant and cited property rate per 1000 population; after controlling for key covariates, the adjusted rate was attenuated (aOR = 1.85, 95% CI = 0.88, 3.88, *p* = 0.10).

**Table 2:** Association between neighborhood vacant and cited property rate and family violence: results of crude and adjusted logistic models

	Crude models		Adjusted models^*a*^	
	Odds ratio	95%CI	Odds ratio	95% CI
**Child maltreatment**				
Model 1: High vacant and cited property rate (dichotomous)	2.11	(1.03, 4.31)	9.20	(1.03, 82.44)
Model 2: Vacant and cited property rate (continuous)	1.01	(1.00, 1.03)	1.05	(1.00, 1.10)
**Intimate partner violence**				
Model 3: High vacant and cited property rate (dichotomous)	2.52	(1.21, 5.25)	1.85	(0.88, 3.88)
Model 4: Vacant and cited property rate (continuous)	1.02	(1.01, 1.04)	1.01	(1.00, 1.02)

^a.^Adjusted for maternal age, education, race, relationship status, ACE history, perceived stress, and anxiety

We examined the mediating role of collective efficacy in both relationships—between vacant and cited property rate and both CM and IPV—and there was no significant indirect effect for collective efficacy in the relation between vacant and cited property and CM (indirect effect estimate = 1.01, *p* = 0.84) or IPV (indirect effect estimate = 1.10, *p* = 0.43).

## Discussion

The results of this study are consistent with the proposed hypothesis that women living in neighborhoods with high rates of vacant and cited property exhibit an increased risk of family violence. Few studies have examined this association even though neighborhood characteristics are important elements of the built environment to examine [8] that have been previously associated with crime and other forms of violence [28]. Children in neighborhoods with higher levels of vacant and cited property were more than twice as likely to experience physical maltreatment, psychological maltreatment, or neglect at 12 months of age, than those living in neighborhoods with lower levels. Similarly, women living in neighborhoods with high levels of vacant and cited property were two and a half times as likely to respond that they had experienced IPV, compared with women living in neighborhoods with lower levels. Therefore, we can conclude that women living in neighborhoods in our study area with more vacant and cited property experience higher rates of family violence. Although we also hypothesized that collective efficacy might help to explain the associations between neighborhood conditions and family violence, when we tested this potential pathway, we observed no significant indirect effects in this sample, although this may be due to a small sample size.

While results for CM remained significant after adjustment for key covariates, IPV results were no longer statistically significant, suggesting more of a connection between neighborhood vacant and cited properties and CM than for IPV. CM measures included both abuse and neglect, whereas IPV measures only included questions related to abuse. Research suggests a strong association between neighborhood socioeconomic factors as stressors and CM and the neighborhood context may play a more direct role on a mother’s ability to safely parent offspring [12]. While neighborhood context has similarly been found to influence the prevalence of IPV, individual characteristics such as age, relationship status, and prior abuse experiences also act as risk or protective factors [8]. Thus, it is possible these maternal level covariates had a stronger influence on IPV than CM. The inclusion of neglect as one of the measures for CM may have further strengthened CM results, given the relationship between neighborhood socioeconomic status and child neglect [7] and the link between neighborhood socioeconomic status and disorder [11].

This study is focused on characteristics of the built environment, so it is important to contextualize our findings within a broader exploration of the root causes of vacant and cited properties, namely, targeted, and chronic disinvestment in community well-being. The built environment is impacted by broader societal factors, such as structural inequity and racism [29]. Historically, community disinvestment has disproportionately impacted communities with higher populations of people of color [30]. Additionally, although Black women made up 65% of this study’s overall sample, 83% of them lived in neighborhoods with high rates of vacant and cited property. The overrepresentation of people of color living in physically disordered neighborhoods in this study reflects the influence of structural inequities including redlining, chronic disinvestment, and racial segregation [30]. Moreover, this specific manifestation of structural inequities contributes to social and health disparities, including the risk of exposure to violence, among people of color [31]. Further, a substantial proportion of participants living in neighborhoods with high vacant and cited property had less than a high school education (35%) or an annual household income of $24,999 or less, both of which are also social determinants of health outcomes [32].

While this study provides important and novel insights into the connection between the neighborhood environment and family violence, it is subject to some notable limitations. The study design cannot demonstrate a causal relationship between neighborhood vacant and cited property and family violence, and there may be unidentified confounders that contribute to the associations identified. Further investigation is needed to understand if and how additional factors at the neighborhood level relate to rates of family violence. Exploration of the potential influences of neighborhood incarceration rates and over policing [33], and the presence of certain structures in the community, such as the density of liquor stores [34], and/or the absence of structures in the built environment (e.g., supportive community institutions) may all play a role. Additionally, given that neighbors may respond differently to safety issues such as family violence [16, 17], research that tests tailored measures for social control and cohesion that may be more relevant to family violence is needed. Further, it is unknown whether the results are generalizable to a broader population, due to a small sample size and sociodemographic characteristics that may be specific to New Orleans. Furthermore, there is the potential for selection bias given the loss to follow-up over the study, with only 64% of the sample with data collected at 12 months. However, women lost to follow-up over the study period did not differ from those who remained in terms of sociodemographic factors, neighborhood conditions, or IPV at baseline. We also had to average data across years given the declining sample size over the course of the follow-up period. There is also a potential for social desirability bias and underreporting for both IPV and CM, due to the legal and social risks, stigma, and trauma associated with reporting family violence, as well as self-report bias. Also, given a neighborhood-based exposure, there may be the potential for spatial autocorrelation which could also impact the findings. However, using a k-neighbor definition of neighborhood (given the lack of full census tract contiguity in the sample), we did not find evidence of spatial autocorrelation based on the Moran’s I statistic (which was not significant). Finally, the measurement of vacant and cited properties with administrative data has certain limitations, as there can be a lack of equitable enforcement of vacant and abandoned property violations and citations and the range of code violations that may be included in enforcement data given the range of conditions that may be cited by a particular municipality. We also did not have information on the frequency of citations by parcel, which could be an important marker for ongoing violations that could have a greater impact on the outcomes. Alternative measures of vacant and cited properties could be explored in future research [35].

## Conclusion

Family violence occurs in the context of deeply rooted structural and social inequities that are reflected in the built environment. The findings from this study contribute to the research literature demonstrating a connection between the built environment, specifically neighborhood vacant and cited property, and family violence, including CM and IPV. Traditionally, many family violence prevention and intervention efforts have focused on interventions at the individual and relational level. Our findings, and those from previous studies [8, 28], support the hypothesis that beyond relational and individual level interventions, neighborhood environment interventions might be alternative avenues for family violence prevention. Community-engaged efforts to improve the built environment by mitigating or maintaining neighborhood vacant and cited property should continue to be a focus of interventions for reducing family violence; however, further research is needed to assess the impact of various elements of disordered neighborhoods on family violence outcomes and to understand mediating and moderating effects to better refine prevention strategies and optimize outcomes. Further, due to high housing costs, the negative effects of gentrification, and the association between concentrated disadvantage and neighborhood disorder, it is also important to consider potential mitigation measures for unintended consequences such as displacement.

## Data Availability

The data that support the findings of this study are available on request from the corresponding author, JMF. The data are not publicly available due to their containing information that could compromise the privacy of research participants.
